# Quality of Life and Socioeconomic Situation of Patients with Hereditary Angioedema in Slovakia

**DOI:** 10.3390/medicina62040705

**Published:** 2026-04-07

**Authors:** Martina Ondrušová, Martin Suchanský, Soňa Vándor Svidová, Katarína Hrubišková, Jana Zelníková, Karolína Vorčáková, Miloš Jeseňák

**Affiliations:** 1 PharmIn Ltd., Karadžičova 16, 82108 Bratislava, Slovakia; 2 Faculty of Public Health, Slovak Medical University, 83303 Bratislava, Slovakia; 3 Centre for Hereditary Angioedema, Allergy-Immunology Outpatient Clinic, 5th Internal Clinic, Faculty of Medicine, Comenius University and Slovak Medical University, 83303 Bratislava, Slovakia; 4 Immunoallergology JZ, Ltd., Faculty Hospital, 01207 Žilina, Slovakia; 5 Department of Dermatovenereology, Jessenius Faculty of Medicine in Martin, Comenius University in Slovakia, University Teaching Hospital, 03659 Martin, Slovakia; 6 National Centre for Hereditary Angioedema, Department of Paediatrics and Adolescent Medicine, Department of Pulmonology and Phthisiology, Jessenius Faculty of Medicine in Martin, Comenius University in Slovakia, University Teaching Hospital, 03659 Martin, Slovakia; 7 Institute of Clinical Immunology and Medical Genetics, Jessenius Faculty of Medicine in Martin, Comenius University in Slovakia, University Teaching Hospital, 03659 Martin, Slovakia

**Keywords:** hereditary angioedema, quality of life, angioedema quality of life questionnaire, angioedema control test, hospital anxiety and depression scale, socioeconomic situation, indirect costs

## Abstract

*Background and Objectives*: Hereditary angioedema (HAE) represents a specific form of life-threatening inborn errors of immunity. Current guidelines recommend regular assessment of the disease burden, disease control and quality of life. This study describes the profile of HAE patients in Slovakia, disease control, quality of life, states of anxiety and depression, and socioeconomic situation. *Materials and Methods*: We used a set of standardized questionnaires—AE-QoL, AECT, HADS and Socioeconomic Status Questionnaire, and a non-standardized questionnaire—to describe the characteristics of the population. *Results*: We collected data on 56.44% (57 out of 101) of HAE adult patients registered in Slovakia. Moderate to severe HAE was present in 61.40% of patients; 73.68% were on long-term prophylactic treatment; and 19.30% received rescue treatment due to an acute HAE attack during the last 4 weeks. Most patients achieved lower AE-QoL scores, indicating a good quality of life. The AECT score indicated well-controlled disease in 91.23% of patients. Anxiety and/or depression scores were higher than normal in 17.54% of patients. Patients with HAE earned less than the average population, but most of them were economically active with relatively low rates of presenteeism and absenteeism. Only a minority of patients used social system benefits. Patients were exclusively cared for by relatives. *Conclusions*: The QoL scores achieved in all three standardized questionnaires indicate a good quality of life of HAE patients in Slovakia, which is associated with a high and specialized standard of care. Anxiety and/or depression were present in 17.54% of patients. Direct patients costs and social care costs are low, but there is an indirect socioeconomic burden on patients and their families.

## 1. Introduction

Hereditary angioedema (HAE) is a rare hereditary disease characterized by sudden, severe, life-threatening attacks of skin and submucous tissue swelling caused by increased vascular permeability due to overproduction of bradykinin [1]. It is a heterogeneous disease with a complex pathophysiology and many of the affected are unaware of their diagnosis. Attacks can be triggered by emotional stress, dental treatment, infection, surgery, injury, medication, menstruation or alcohol, but the trigger is often unknown [2,3].

Episodes of swelling last 1–5 days on average. Edema can affect different parts of the body, including the face, upper respiratory tract, limbs, or gastrointestinal tract [4]. Laryngeal angioedema, i.e., swelling of the upper respiratory tract, is the most severe and potentially fatal clinical manifestation, as it can lead to airway obstruction and asphyxia.

Recently, an analysis of the National Registry of HAE Patients in Slovakia was published, identifying a total of 126 patients (101 adult patients; 80.16%) with an estimated prevalence and incidence of 1:41,280 and 1:1,360,000, respectively. Among them, 12 novel, previously undescribed mutations were found [5]. The frequency of attacks varies widely [6]. In Slovakia, the frequency of more than 10 attacks per year was recorded in 7.14% of patients, 6–10 attacks in 8.16%, five and less attacks in 84.69% of patients, and 38.78% of patients experienced laryngeal swelling during their lifetime [7].

The management of HAE is based on prophylaxis (long-term or short-term) and treatment of acute attacks (rescue treatment). Long-term prophylaxis focuses on preventing or reducing the frequency and severity of attacks, whereas short-term prophylaxis is indicated to prevent attacks in anticipated high-risk situations, such as surgery, intubation, dental surgery, etc. [6]. Prophylactic treatment usually includes androgens (e.g., danazol, stanozolol), antifibrinolytics (e.g., tranexamic acid, epsilon-aminocaproic acid), human C1-INH concentrate (intravenous or subcutaneous), berotralstat or lanadelumab. However, current guidelines recommend only pdC1-INH, lanadelumab or berotralstat for the long-term prophylaxis in the first line of the treatment [2,8,9,10].

The aim of the rescue treatment is to reduce the severity and duration of angioedema. The treatment is significantly more effective if it is given at the onset of an attack within the first few hours [11]. An effective rescue treatment is the intravenous administration of a concentrate of human C1-INH in the form of a plasma derivative as well as a recombinant human C1-INH protein. Another option is subcutaneous icatibant (bradykinin B2 receptor antagonist) [10,12].

Recurrent angioedema attacks are associated with a significant reduction in quality of life for both patients and their relatives in all areas of life [13]: limitation of daily activities, fear of having an attack due to its unpredictability, increased absence from work or school, as well as need for nursing care [2,14].

Despite these facts, there is relatively little data on quality of life in HAE patients in the literature, and local data from Slovakia are not available at all. Indirect costs associated with HAE are difficult or even impossible to assess from hard data due to the lack of a clear classification of HAE.

The aim of this study was to describe the current profile of patients with HAE in Slovakia (demographics, categorization of HAE, therapy), to assess disease control, to determine their quality of life, to detect states of anxiety and depression and to describe their socioeconomic situation (presenteeism, absenteeism, social benefits, need for informal care, individual costs and limitations due to HAE).

## 2. Materials and Methods

### 2.1. Study Design

We present a quantitative cohort study that retrospectively evaluated the medical and socioeconomic situation of patients over a specified period. The study was descriptive only and did not evaluate specific treatment outcomes. The primary objective was a descriptive characterization of Slovak HAE patients, including demographic data, quality of life and socioeconomic status, as the set of collected information offers a unique opportunity to analyze a general cohort of patients from a single European country, without the need to extrapolate the results to larger cohorts. Moreover, the aim was to cover as large a proportion of the HAE patient population as possible. The secondary objective was an exploratory analysis of differences in quality of life between different patient groups.

### 2.2. Study Population

The monitored Slovak HAE patients were treated in two centres: 1. National Reference Centre for Hereditary Angioedema, University Hospital in Martin; 2. Centre for Hereditary Angioedema, 5th Department of Internal Medicine, Faculty of Medicine, Comenius University Bratislava and University Hospital in Bratislava. The inclusion criteria were slightly different for individual questionnaires; therefore, the study population was divided into two groups: A and B. Inclusion criteria, identical for both cohorts (A and B), were as follows: definitively diagnosed HAE at any time in the past; signed consent for the processing of personal data for the purpose of participation in the presented study; citizenship of the Slovak Republic. Extended inclusion criteria for the Socioeconomic Status Questionnaire (cohort B): the patient fulfils the local conditions for any form of work performance (according to the Labour Code—Act No. 311/2001 Coll., § 11, section 2 [15]), regardless of whether they have actually worked—specifically, the patient has reached the age of 15 and at the same time has completed compulsory schooling. All patients met the criteria for completing questionnaires A and B. Exclusion criteria for all questionnaires were the diagnosis of another serious disease in the terminal stage, severe comorbid conditions due to HAE and death of the patient.

### 2.3. Primary and Secondary Outcomes

The primary outcomes of the study were to collect data on quality of life, disease control and psychological condition of HAE patients in Slovakia. The secondary outcomes were to assess the socioeconomic status of the patients, their social benefits and the need for informal care.

### 2.4. Questionnaires Specification

The data collection consisted of five questionnaires (3 standardized and the rest non-standardized), each focusing on specific target areas.

### 2.5. Basic Patient Profile Questionnaire

The non-standardized questionnaire focused on the description of the patient profile and HAE treatment and contained 17 questions [16]. The assessment period was specifically defined for each question: from the date of diagnosis, retrospectively for the last 4 weeks, or up to the date of completion of the questionnaire. The HAE severity parameter for the last year was approved by the study validators before the start of the study according to Prior et al. [17].

### 2.6. Angioedema Quality of Life Questionnaire (AE-QoL)

AE-QoL is an angioedema-specific quality of life (QoL) questionnaire suitable for the adult population (>18 years). The questionnaire consists of 17 items divided into four domains, Functioning, Fatigue/Mood, Fears/Shame and Nutrition, and has a recall period of 4 weeks. Domain scores and total scores range from 0 to 100, with higher scores indicating greater QoL impairment. The AE-QoL version used was the Angioedema Quality of Life Questionnaire—Slovakian Version 2017 (provided with the official Slovakian translation).

### 2.7. Angioedema Control Test (AECT)

The standardized AECT questionnaire is suitable for the adult population (>18 years) with all forms of recurrent angioedema [18,19]. The questionnaire monitors disease control retrospectively, with a recall period of 4 weeks. It contains 4 questions with the score ranging from 0 to 16. A cut-off score of <10 points corresponds to poorly controlled disease [18,19]. The AECT 4-week [20] recall version was used in this study. The questionnaire was translated into Slovak by the study implementers for the purpose of this research. The translation was not subjected to a formal forward–backward translation process, cognitive debriefing, or independent psychometric validation in the Slovak population. Therefore, although the original instrument has been formally validated and the cut-off value of ≥10 points corresponds to well-controlled disease in validated language versions, the Slovak version used in this study should be considered exploratory.

### 2.8. Hospital Anxiety and Depression Scale (HADS)

The standardized HADS questionnaire consists of 14 questions and is suitable for any population without restrictions. The HADS is a simple total score divided into two separately scored scales: anxiety (A-scale) and depression (D-scale) [21]. For both separately, the score ranges from 0 to 21. The HADS score is divided into interpretation categories: normal (0–7), mild (8–10), moderate (11–14) and severe (15–21). The recall period was 7 days. We used the HADS—Slovakia/Slovak—Version of 13 March 2017 (updated 23 June 2021)—Mapi (with an official Slovak translation) [22,23,24].

### 2.9. Socioeconomic Status Questionnaire (SESQ)

A non-standardized questionnaire called the Evaluation of the Socioeconomic Situation of Patients with HAE consisted of 37 questions. It was developed by Pharm-In, Ltd. and study validators. It is suitable for patients with any disease who have reached 15 years of age and have completed compulsory education in accordance with the Labour Code—Act No. 311/2001 Coll., § 11 section 2. The SESQ is not a scoring questionnaire; it is based on a system of sorting categories and identification of the occurrence of individual facts in the monitored population. The evaluation period was 12 months retrospectively. The version used was SESQ v2022.06 (prepared in the Slovak language).

### 2.10. Data Collection

The data collection period was 1 July–30 September 2022. The final analysis of the data was performed during the period May–June 2025. During this period, the necessary data were extracted from the patients’ medical records, and the patients completed the pre-designed online forms with the assistance of the physician during their medical examinations [25]. Demographic data included age (current and at diagnosis), sex, type and form of HAE, current prophylaxis status, use of rescue treatment in the last 4 weeks and number of acute HAE attacks in three time periods (last 7 days/4 weeks/12 months). An acute HAE attack was defined as the sudden onset of angioedema symptoms in the subcutaneous and/or submucosal compartment at one or more body sites. The questionnaires did not contain any personal data that could be used to identify patients. The study was approved by the Ethics Committee of University Hospital Martin (Martin, Slovakia)—No. EK UNM24/2022, 12 April 2022.

### 2.11. Data Analysis Considerations

All patients (100%) answered all questions on all questionnaires.

All analyses were descriptive and exploratory in nature. Categorical variables were summarized using absolute and relative frequencies. Continuous variables were described using number of observations, mean, standard deviation (SD), median, interquartile range (IQR), and range. As most variables were not normally distributed, group comparisons were performed using non-parametric tests (Mann–Whitney U test or Kruskal–Wallis test when appropriate). When the Kruskal–Wallis test indicated a global difference between groups, no post hoc pairwise comparisons were performed due to the small sample size and very small subgroup counts (e.g., severe HAE, *n* = 2). Therefore, the results of the Kruskal–Wallis test should be interpreted as exploratory global comparisons only.

Given the exploratory character of the study and the limited sample size inherent to a rare disease cohort (*n* = 57), no formal adjustment for multiple testing (e.g., Holm or false discovery rate correction) was applied. All reported *p*-values should therefore be interpreted as exploratory. Statistical findings were evaluated in the context of clinical relevance, effect direction, and consistency across related outcome measures rather than on isolated statistical significance.

Data were processed and analyzed using Microsoft Excel [Microsoft 365 Business Standard] and STATISTICA 14 [26].

## 3. Results

### 3.1. Characteristics of Patient’s Population

According to the Registry of HAE Patients in Slovakia, the number of all living HAE patients as of 31 December 2021 was 126. The calculated prevalence was 1:43,000. The adult population was represented by 101 patients (80.16%) [27]. In this study, data was collected from *n* = 57 adult patients, representing 56.44% of adult patients registered in the HAE Patient Registry. Descriptive characteristics of the enrolled population are summarized in Table 1. Females predominated in the study: 56.14% (*n* = 32) vs. 43.86% males (*n* = 25). The median age at diagnosis was 23.52 years (IQR 16.42–32.48) and 44.53 years (IQR 33.33–55.47) at the time questionnaire completion. More than a half of the patients (61.40%; *n* = 35) had a moderate to severe form of HAE. More than half of the patients were receiving prophylactic treatment (73.68%; *n* = 42) (Table 1). Rescue treatment in the last 4 weeks was given to 19.30% (*n* = 11) of all patients in the study (Table 1).

### 3.2. QoL Questionnaires

Due to the asymmetry in the distribution of patients on individual QoL scores (and sorting subgroups), interpretation of QoL scores via median with IQR is recommended.

#### 3.2.1. AE-QoL Standardized Questionnaire

**Figure 1:**
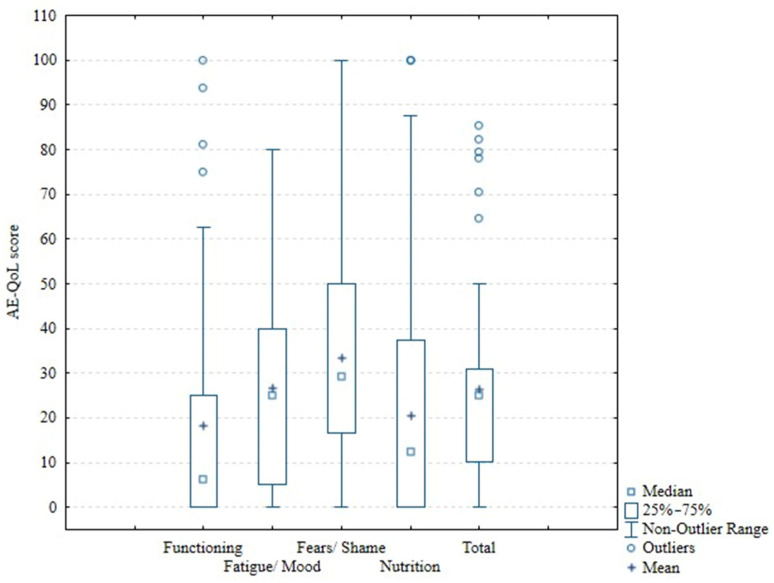
AE-QoL score.

Most patients achieved lower AE-QoL scores, which is desirable and indicates better quality of life. The median total AE-QoL score for the whole population was 25 (IQR 10.29–30.88) (Table 2, Figure 1). There were statistically significant differences in total scores between patients with different uses of prophylaxis and use of rescue treatment (Table 2).

#### 3.2.2. AECT Standardized Questionnaire

The total AECT score reached a median of 14.00 (IQR 12–15) in the population (Table 3). Most patients in the total population (91.23%) achieved an AECT score of 10 or more, indicating a well-controlled disease, regardless of sex, form of HAE and use of prophylaxis. The only statistically significant differences were between patients who had had an acute attack in the last 4 weeks and those who had not, and between patients who had received rescue treatment in the last 4 weeks and those who had not. Patients who used rescue treatment in the last 4 weeks achieved a median score of 11 (IQR 8–12), which is 1 point above the cut-off value for sufficiently controlled disease (Table 3).

#### 3.2.3. HADS Standardized Questionnaire

**Figure 2:**
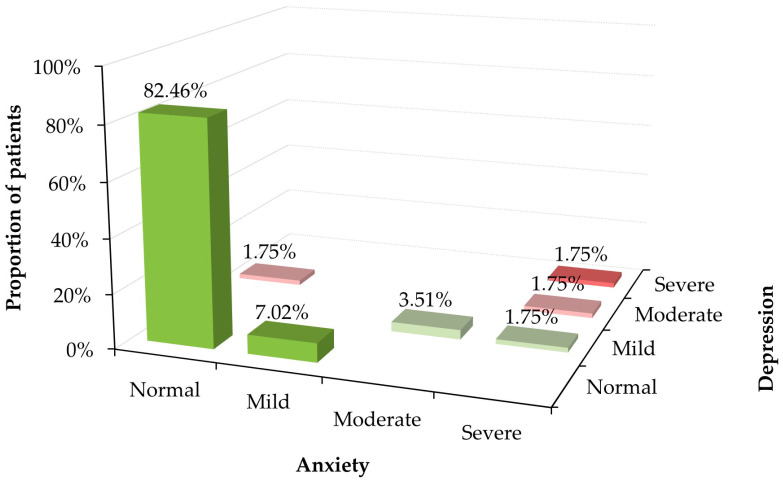
Distribution of patients according to Hospital Anxiety and Depression Scale (HADS).

Lower HADS scores indicate good psychological condition in most patients (Table 4). The majority (82.46%) achieved normal scores (Figure 2). Statistically significant differences were only between anxiety and depression scores of patients with and without at least one acute attack during the last 7 days, but only two patients had these acute attacks, and their anxiety or depression were mild or severe. However, the scores can be considered normal for all another groups. Most cases of moderate to severe anxiety and depression were observed in patients on prophylaxis.

### 3.3. Number of Acute Attacks

In the last 12 months 14.04% of our patients had >10 acute attacks, 10.53% had 6–10 acute attacks and 73.68% had 0–5 acute attacks. In 1.75% of patients the number of acute attacks was unknown . There were significant differences between patients with and without rescue treatment and between patients with different forms of HAE (Table 5).

### 3.4. Socioeconomic Situation of HAE Patients in the Last 12 Months (Questionnaire B)

All patients who met the additional inclusion criterion (*n* = 57) participated in the online questionnaire B.

Most patients in the study do not receive most social benefits and are relatively economically active. The study covers a wide range of employment statuses: students, parents on maternity/parental leave, recipients of various pensions, full-time and part-time employees and self-employed.

Among the economically active population, the calculated average gross monthly income of €1140.44 was lower than the country’s average nominal monthly income in 2022 (€1304.00) [28].

### 3.5. Economic Activity, Declined Work Performance, Presenteeism, Absenteeism and Activity Limitation Due to HAE in the Last 12 Months

**Figure 3:**
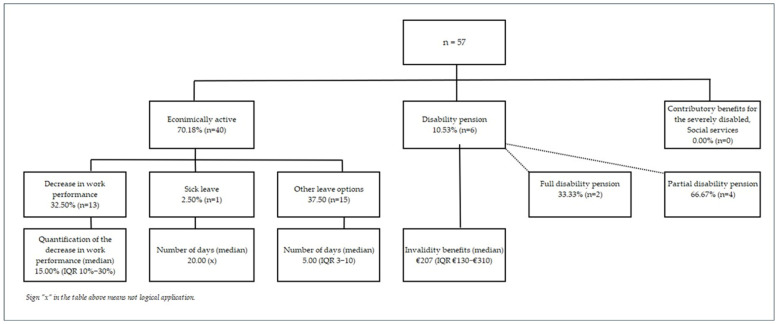
Economic activity and declined work performance due to HAE.

In the last 12 months 70.18% of patients were at least partially economically active (Figure 3). There were proportionally more economically active patients in the group without prophylaxis (80.00% of all patients without prophylaxis) than in the group with prophylaxis (66.12% of all patients with prophylaxis).

A decrease in work performance due to HAE in the last 12 months was reported by 32.50% of economically active patients (Figure 3), of whom 92.31% were on prophylaxis. The median reduction in work performance was 15.00% (IQR 10.00–30.00%).

Only one patient took sickness benefits cumulative for 20 days in the last 12 months (Figure 3). Other forms of leave due to HAE (holidays, sick days, unpaid leave, etc.) were used by 37.50% of patients (median = 5 days; IQR 3–10 days). Among patients with prophylaxis (*n* = 28), 42.86% took a median of 4.5 days off work (IQR 3–7). In patients without prophylaxis (*n* = 12), 25.00% took a median of 15 days off work (IQR 4–30).

More than half (56,14%, *n* = 32) of the patients had to limit their activities in the last 12 months due to HAE, of whom 75.00% reported limitations in their leisure activities: 40.63% in work; 37.50% in travel; 3,13% in sports; 3,13% in routine housework and childcare.

### 3.6. Disability Pension and Contributory Benefits for the Severely Disabled Due to HAE in the Last 12 Months

Of the patients in our study, 10.53% were receiving some kind of invalidity benefits, of which 33.33% were receiving full invalidity benefits and 66.67% partial invalidity benefits (Figure 3). One patient received partial invalidity benefits due to HAE (16.67%). The median of invalidity benefits was €207/month (IQR €130–€310). None of the patients received contributory benefits for the severely disabled due to HAE in the past 12 months.

### 3.7. Need for Formal and Informal Care Due to HAE in the Last 12 Months

No patient in the study used social services or 24 h residential care in a social service facility in the last 12 months. Of all patients, only 15.79% (*n* = 9) required informal care, and they were cared for by a median of two persons (IQR 1–2 persons) in the last 12 months. A total of 80.00% of patients identified the caregiver as their partner, 50.00% as at least one of their parents and 10.00% as at least one of their children. Caregivers spent a median of 10 h per week (IQR 2–20 h) actively caring for a patient. None of the caregivers had received any financial contributions for the care of a severely disabled person due to patient’s HAE disease, and none of the relatives received any caregiver’s allowance due to patient’s HAE disease in the last 12 months. Most patients (77.78%) needed help with household tasks; 66.67% with shopping care; 55.56% with transport to the doctor; and 33.33% with personal hygiene.

### 3.8. Out-of-Pocket Cost Due to HAE in the Last 12 Months

A total of 87.72% of patients reported zero average co-payments for healthcare due to HAE in the last 12 months (Table 6). All patients in the study had zero co-payments for social care due to HAE. More than a half of them (64.91%) reported zero increase in total costs due to HAE in the last 12 months (median €0, IQR €0–€10). The mean value of €18.28 (SD = 71.16) was driven by one extreme value (Table 6).

### 3.9. Net Monthly Income During the Last 12 Months

The median of the mean net monthly income in the last 12 months was €750.00 (range €174–€10,000). Due to significant asymmetry in the data, outliers, and extreme values for this item, interpretations based on mean values are not recommended. In median, patients with prophylaxis had a lower income (median €700.00; range €174.00–€10,000; *n* = 41) than patients without prophylaxis (median €900.00; range €390.00–€2000.00; *n* = 15).

## 4. Discussion

This study describes the quality of life of HAE patients in Slovakia, including disease control, psychological condition, and socioeconomic situation. It adds data on QoL in Slovak HAE patients to the recently published epidemiological and molecular genetic data in this cohort [5].

It involves *n* = 57 adult patients, which represents more than a half (56.44%) of the general cohort of diagnosed patients with HAE in the Slovak Republic (population of *n* = 5,434,712 in 2022). Beyond confirmed C1-INH-HAE (type I/II), underestimation may still occur, particularly due to diagnostic delays in patients without a positive family history, frequent misdiagnosis as allergic disorders, limited awareness among non-specialists, and the diagnostic complexity of HAE with normal C1-INH. Despite these challenges, in Slovakia the unique centralization of patients into coordinated national expert centers and the existence of a national HAE registry have enabled the capture of a relatively high number of confirmed C1-INH-HAE cases (e.g., *n* = 132; prevalence ~1:41,280), which is comparable to or higher than figures reported in neighbouring countries such as Austria (*n* = 137; prevalence ~1:64,396) and the Czech Republic (national cohort reports ~207 patients [5]. Given the rarity of the disease, available publications of similar nature analyze similar patient cohorts. Aygören-Pürsün et al. [14] analyzed the burden of illness on *n* = 186 HAE patients from Spain (*n* = 58), Germany (*n* = 62) and Denmark (*n* = 44). A Hungarian study [29] analyzed *n* = 125 HAE patients, while the population of Hungary is approximately 1.78 times the population of Slovakia. And Fouche et al. [30] determined the prevalence of depression and anxiety in *n* = 26 US HAE patients.

Descriptive observation shows the following relationship between the three questionnaires: lower AE-QoL scores ≥ higher AECT scores ≥ lower HADS scores. Rather desirable quality of life scores were achieved in all three standardized questionnaires.

This study showed that the AE-QoL scores in Slovak patients were lower than those reported in most foreign studies [10,31,32,33,34,35]. This indicates a good quality of life of Slovak HAE patients due to highly specialized standard care. We did not observe statistically significant differences in AE-QoL scores between men and women, which contradicts the findings of some foreign studies, which observed significantly higher AE-QoL total or domain scores in women [29,34,36], or conversely, in men [33]. Patients with prophylaxis had significantly higher AE-QoL scores than patients without prophylaxis. This finding again contradicts the results of other studies, in which use of prophylaxis led to an improved quality of life [32,37]. Our result can be explained by the fact that in our study, prophylactic treatment was received mostly by patients with moderate to severe forms of HAE (*n* = 35). Patients who required rescue treatment in the last 4 weeks had significantly higher AE-QoL scores than those who did not. We assume that these patients suffered an acute HAE attack during this period. Several studies have shown a positive correlation between the number of attacks during a given period and the AE-QoL scores [10,29,38,39]. Moreover, studies with innovative prophylactic treatment, e.g., lanadelumab, showed a significant and clinically meaningful improvement in AE-QoL very shortly after the initiation of the treatment [40].

Compared with multinational burden-of-illness surveys reporting lower disease control and higher rates of anxiety and depression, our cohort demonstrated more favourable outcomes. These differences may reflect variation in healthcare organization, centralized specialist care, and particularly the relatively high uptake of long-term prophylaxis in our population. In our cohort, 73.68% of patients were receiving long-term prophylaxis, which is higher than in many published European datasets. Greater access to and use of modern prophylactic therapies may contribute to improved disease control and HRQoL, and lower psychological burden. Therefore, cross-country comparisons should be interpreted in light of differences in treatment availability and healthcare system organization. Recent systematic analysis of 65 articles highlighted the importance of early diagnosis and access to the effective treatment to reduce the burden of HAE on patients and society [41].

Significant differences in the AECT score were, as expected, between patients with at least one acute attack in the last 4 weeks and those without, and between patients with rescue treatment in the last 4 weeks and those without. The relationship between decreasing AECT scores and increasing number of attacks has been shown previously [10]. Surprisingly, there was no difference between patients with and without prophylactic treatment, which is not consistent with findings of other authors [32]. Recent studies have demonstrated that AECT is sensitive to clinical change and that a minimal clinically important difference (MCID) has been established (three points of improvement). However, given the cross-sectional design of our study, assessment of responsiveness or MCID-based interpretation was not applicable [42].

The majority of Slovak patients did not suffer from anxiety or depression in the last 7 days. Anxiety and/or depression were present in only 17.54% of patients. The median anxiety score of 3 (IQR 2–6) and median depression score of 2 (IQR 1–4) suggest a good psychological condition of patients in comparison with foreign studies [10,32]. Only five patients had moderate to severe anxiety levels, and one patient had severe depression scores. This patient, on disability pension for anxiety-depressive disorder, had high anxiety (HADS score 18) and high depression (HADS score 19) scores.

Patients receiving long-term prophylaxis had higher AE-QoL scores compared with patients without prophylaxis. This finding should not be interpreted as a negative effect of prophylactic treatment. In our cohort, prophylaxis was predominantly administered to patients with moderate to severe disease, reflecting clinical indication rather than random allocation. Therefore, the observed differences most likely represent confounding by indication, with patients on prophylaxis having a higher baseline disease burden.

Given the cross-sectional design and limited sample size, multivariable adjustment was not performed, as modelling with multiple covariates would be statistically unstable and prone to overfitting. Consequently, comparisons between prophylaxis and non-prophylaxis groups are descriptive and exploratory. No causal inference regarding treatment effect can be made.

Evidence from larger international cohorts suggests that, after appropriate adjustment for disease severity and other confounders, long-term prophylaxis is associated with improved disease control and better patient-reported outcomes. Our findings should therefore be interpreted within the context of baseline disease severity rather than as an effect of treatment itself.

Phase 3 and extension studies of modern prophylactic agents have consistently demonstrated clinically meaningful improvements in HRQoL and disease control, supporting the plausibility of the favourable outcomes observed in a cohort with high prophylaxis uptake.

Our observations on the socioeconomic situation of HAE patients showed that most patients in the study do not receive most social benefits. They are relatively economically active, with relatively low presenteeism and absenteeism compared with other studies [10,14]. Patients with prophylaxis took more days off work than those without prophylaxis, which can be explained by the severity of the underlying disease, but their absence from work was much shorter.

Concerning the total costs of HAE, 35.09% of patients reported more than zero total cost of the disease and additional payments for healthcare, even though all treatments for HAE are fully covered by health insurance. Social care costs were absent, probably due to the relatively young median age of patients and informal care provided by relatives. These findings suggest that even if direct out-of-pocket costs to patients and social care costs to society are low, there is an indirect socioeconomic burden on patients and their families in the form of negative impact on career or education, lost productivity, lost time from unpaid work or foregone leisure time.

The presented study has some limitations. As the aim of this study was to describe the current profile of patients with HAE in Slovakia, we used a cross-sectional design, which captures data at a single time-point but cannot track changes over time. An additional limitation concerns the use of the Angioedema Control Test (AECT). Although AECT is a validated patient-reported outcome instrument with established psychometric properties, including sensitivity to change and a defined cut-off for well-controlled disease, the Slovak version used in our study did not undergo formal cross-cultural validation. The translation was prepared by the study implementers and was not subjected to forward–backward translation procedures or independent validation in the Slovak population. Therefore, AECT-based findings should be interpreted with caution and considered supportive rather than definitive. To mitigate this limitation, conclusions regarding disease control were triangulated with independently collected clinical indicators (attack frequency and rescue treatment use) and with AE-QoL results obtained using a formally validated Slovak version.

An important limitation relates to multiple exploratory comparisons performed across questionnaire domains and patient subgroups. As no formal correction for multiple testing was applied, the risk of type I error cannot be excluded. Reported *p*-values should therefore be interpreted cautiously and considered hypothesis-generating rather than confirmatory. Given the limited sample size, formal multiplicity adjustments would likely have increased type II error and reduced interpretability in this rare disease cohort. Finally, comparisons between patients receiving and not receiving long-term prophylaxis must be interpreted in light of confounding by indication. Patients on prophylaxis predominantly represented individuals with moderate to severe disease. Due to the cross-sectional design and limited sample size, multivariable adjustment was not performed, as this would risk statistical instability and overfitting. Consequently, associations between prophylaxis and patient-reported outcomes should not be interpreted as causal treatment effects but rather as descriptive differences within the national cohort.

An important contextual limitation is the relatively high proportion of patients receiving long-term prophylaxis in our cohort (73.68%), which exceeds proportions reported in several multinational and European datasets. Higher uptake of prophylactic therapy may contribute to the improved disease control, better HRQoL, and lower psychological burden observed in our population. Consequently, generalizability of these findings to settings with lower access to or uptake of long-term prophylaxis may be limited. To estimate the long-term impact of HAE on quality of life and socioeconomic status of the patients, further research will be needed.

## 5. Conclusions

Hereditary angioedema represents a lifetime and life-threatening severe disease with significant impact on all aspects of daily life for patients and their families. Monitoring disease burden and quality of life could help to modify and personalize the treatment strategy. Various psychological disorders and problems, identified in a significant proportion of patients, should be considered in the complex management of the disease.

## Figures and Tables

**Table 1:** Characteristics of the patient population.

**Characteristics** **of Patient Population**	
Number of all living HAE patients in Slovakia (as of 31.12.2021)	126 (100%)
Only adult patients	101 (80.16%)
Number of living HAE patients in the study (01.07.2022 to 30.09.2022) ^1^	57 (56.44%)
Questionnaire A	57
Questionnaire B	57
Distribution by sex (number of patients)	57 (100.00%)
Females	32 (56.14%)
Males	25 (43.86%)
Age of the patients in years: median (range)	
Age at diagnosis	23.52 (16.42–32.48)
Age at completing the questionnaire	44.53 (33.33–55.47)
Type of HAE (number of patients)	57 (100.00%)
I. type	51 (89.47%)
II. type	6 (10.53%)
Severity of HAE (number of patients)	57 (100.00%)
Asymptomatic form	3 (5.26%)
Mild form	19 (33.33%)
Moderate form	33 (57.89%)
Sever form	2 (3.51%)
Current prophylactic status (number of patients)	57 (100.00%)
Patients on prophylactic treatment	42 (73.68%)
Patients without prophylactic treatment	15 (26.32%)
Patients with Rescue treatment during the last 4 weeks (number of patients)	57 (100.00%)
Yes	11 (19.30%)
No	46 (80.70%)
At least 1 acute attack during the last 7 days (number of patients)	
Yes	2 (3.51%)
No	52 (91.23%)
Unknown ^2^	3 (5.26%)
At least 1 acute attack during the last 4 weeks (number of patients)	
Yes	11 (19.30%)
No	44 (77.19)
Unknown ^2^	2 (3.51%)
At least 1 acute attack during the last 12 months (number of patients)	
Yes	39 (68.42%)
No	17 (29.82%)
Unknown ^2^	1 (1.75%)

^1^ The data are for different time periods, so the proportion given is only a rough estimate and does not reflect the real situation. For example, some patients may have died in the meantime and others may have been newly diagnosed. ^2^ “Unknown” is not included in further analysis, where this parameter is considered to be.

**Table 2:** AE-QoL score.

AE-QoL Score	AE-QoL Domains				Total AE-QoL
	Functioning	Fatigue/Mood	Fears/Shame	Nutrition	
**Females**					
Patients with record, *n*	32	32	32	32	32
Median (IQR)	0 (0–25)	30 (10–42.5)	29.17 (22:92–50)	12.5 (0–31.25)	26.47 (9.56–35.29)
**Males**					
Patients with record, *n*	25	25	25	25	25
Median (IQR)	12.5 (0–25)	20 (5–35)	25 (12.5–37.5)	12.5 (0–37.5)	20.59 (10.29–29.41)
***p*** **-value (Mann–Whitney U Test)**	0.5768	0.2703	0.3168	0.9252	0.5090
At least 1 acute attack during the last 4 weeks *					
Patients with record, *n*	11	11	11	11	11
Median (IQR)	18.75 (0–37.5)	30 (5–40)	37.5 (12.5–54.17)	25 (0–37.5)	29.41 (11.76–45.59)
No acute attack during the last 4 weeks *					
Patients with record, *n*	44	44	44	44	44
Median (IQR)	0 (0–25)	20 (2.5–40)	25 (16.67–39.58)	0 (0–25)	24.26 (8.82–29.41)
***p*** -value (Mann–Whitney U Test) *	0.1853	0.5811	0.4725	0.2380	0.3484
**Prophylactic treatment during the last 4 weeks**					
Patients with record, *n*	42	42	42	42	42
Median (IQR)	12.5 (0–31.25)	27.5 (15–40)	29.17 (20.83–50)	12.5 (0–37.5)	27.21 (11.76–39.71)
**Without prophylactic treatment during the last 4 weeks**					
Patients with record, *n*	15	15	15	15	15
Median (IQR)	0 (0–25)	15 (0–35)	16.67 (12.5–33.33)	0 (0–25)	16.18 (4.41–27.94)
***p*** **-value (Mann–Whitney U Test)**	0.1665	0.1978	**0.0325**	0.532	**0.0489**
**With rescue treatment during the last 4 weeks**					
Patients with record, *n*	11	11	11	11	11
Median (IQR)	25 (12.5–37.5)	35 (25–40)	37.5 (29.17–54.17)	25 (12.5–37.5)	29.41 (20.59–45.59)
**Without rescue treatment during the last 4 weeks**					
Patients with record, *n*	46	46	46	46	46
Median (IQR)	0 (0–25)	20 (0–40)	25 (16.67–41.67)	0 (0–25)	21.32 (7.35–29.41)
***p*** **-value (Mann–Whitney U Test)**	**0.0227**	0.1189	0.0604	**0.0484**	**0.0233**
**Asymptomatic form HAE**					
Patients with record, *n*	3	3	3	3	3
Median (IQR)	0 (0–25)	35 (15–40)	29.17 (16.67–50)	12.5 (0–37.5)	26.47 (17.65–27.94)
**Mild form HAE**					
Patients with record, *n*	19	19	19	19	19
Median (IQR)	6.25 (0–25)	25 (0–55)	29.17 (12.5–54.17)	12.5 (0–50)	26.47 (7.35–41.18)
**Moderate form HAE**					
Patients with record, *n*	33	33	33	33	33
Median (IQR)	6.25 (0–25)	25 (10–40)	25 (20.83–41.67)	12.5 (0–37.5)	25 (8.82–30.88)
**Severe form HAE**					
Patients with record, *n*	2	2	2	2	2
Median (IQR)	6.25 (0–12.5)	20 (5–35)	20.83 (12.5–29.17)	12.5 (0–25)	16.18 (11.76–20.59)
***p*** **-value (Kruskal–Wallis Test)**	0.8505	0.9238	0.8676	0.9607	0.9428

Statistically significant *p*-value (α < 0.05) is highlighted in bold. * Patients with unknown number of acute attacks are not included.

**Table 3:** AECT score.

AECT Score	
**All patients**	
Patients with record, *n*	57
Median (IQR)	14 (12–15)
**Females**	
Patients with record, *n*	32
Median (IQR)	14 (12–15.5)
**Males**	
Patients with record, *n*	25
Median (IQR)	14 (12–15)
***p*** **-value (Mann–Whitney U Test)**	0.8515
At least 1 acute attack during the last 4 weeks *	
Patients with record, *n*	11
Median (IQR)	12 (6–15)
No acute attack during the last 4 weeks *	
Patients with record, *n*	44
Median (IQR)	14 (13–15.5)
***p*** -value (Mann–Whitney U Test) *	**0.0159**
**Prophylactic treatment during the last 4 weeks**	
Patients with record, *n*	42
Median (IQR)	14 (12–15)
**Without prophylactic treatment during the last 4 weeks**	
Patients with record, *n*	15
Median (IQR)	14 (13–15)
***p*** **-value (Mann–Whitney U Test)**	0.8472
**With rescue treatment during the last 4 weeks**	
Patients with record, *n*	11
Median (IQR)	11 (8–12)
**Without rescue treatment during the last 4 weeks**	
Patients with record, *n*	46
Median (IQR)	14.5 (13–16)
***p*** **-value (Mann–Whitney U Test)**	**0.0002**
**Asymptomatic form HAE**	
Patients with record, *n*	3
Median (IQR)	14 (13–15)
**Mild form HAE**	
Patients with record, *n*	19
Median (IQR)	13 (10–15)
**Moderate form HAE**	
Patients with record, *n*	33
Median (IQR)	14 (12–16)
**Severe form HAE**	
Patients with record, *n*	2
Median (IQR)	14 (13–15)
***p*** **-value (Kruskal–Wallis Test)**	0.7051

Statistically significant *p*-value (α < 0.05) is highlighted in bold. * Patients with unknown number of acute attacks are not included.

**Table 4:** HADS score. The effect of rescue treatment was not investigated as rescue treatment during the last 7 days was not recorded.

HADS Score	Anxiety	Depression
**All patients**		
Patients with record, *n*	57	57
Median (IQR)	3 (2–6)	2 (1–4)
**Females**		
Patients with record, *n*	32	32
Median (IQR)	3 (2–5.5)	1 (1–3)
**Males**		
Patients with record, *n*	25	25
Median (IQR)	4 (1–7)	2 (1–5)
***p*** **-value (Mann–Whitney U Test)**	0.6978	0.1283
At least 1 acute attack during the last 7 days *		
Patients with record, *n*	2	2
Median (IQR)	16.5 (15–18)	13.5 (8–19)
No acute attack during the last 7 days *		
Patients with record, *n*	52	52
Median (IQR)	3.5 (2–6)	2 (1–4)
***p*** -value (Mann–Whitney U Test) *	**0.0188**	**0.0271**
**Prophylactic treatment during the last 7 days**		
Patients with record, *n*	42	42
Median (IQR)	4 (2–6)	2 (1–4)
**Without prophylactic treatment during the last 7 days**		
Patients with record, *n*	15	15
Median (IQR)	3 (1–6)	1 (0–5)
***p*** **-value (Mann–Whitney U Test)**	0.3385	0.3864
**Asymptomatic form HAE**		
Patients with record, *n*	3	3
Median (IQR)	4 (4–6)	5 (1–11)
**Mild form HAE**		
Patients with record, *n*	19	19
Median (IQR)	3 (1–8)	2 (1–5)
**Moderate form HAE**		
Patients with record, *n*	33	33
Median (IQR)	4 (2–6)	2 (1–3)
**Severe form HAE**		
Patients with record, *n*	2	2
Median (IQR)	1 (0–2)	1.5 (1–2)
***p*** **-value (Kruskal–Wallis Test)**	0.3923	0.5576

Statistically significant *p*-value (α < 0.05) is highlighted in bold. * Patients with unknown number of acute attacks are not included.

**Table 5:** Number of acute attacks during a specified time period.

Number of Acute Attacks During the Specific Period of Time	Last 7 Days	Last 4 Weeks	Last 12 Months
**All patients**			
Patients with record, *n*	54	55	56
Mean (SD)	0.04 (0.19)	0.24 (0.51)	4.7 (7.17)
Median (IQR)	0 (0–0)	0 (0–0)	2.5 (0–5)
**Females**			
Patients with record, *n*	31	31	32
Median (IQR)	0 (0–0)	0 (0–0)	3 (1–6.5)
**Males**			
Patients with record, *n*	23	24	24
Median (IQR)	0 (0–0)	0 (0–0)	1 (0–3.5)
***p*** **-value (Mann–Whitney U Test)**	0.8515	1.0000	0.0824
**prophylactic treatment during the last 7 days**			
Patients with record, *n*	41	NA	NA
Median (IQR)	0 (0–0)	NA	NA
**Without prophylactic treatment during the last 7 days**			
Patients with record, *n*	13	NA	NA
Median (IQR)	0 (0–0)	NA	NA
***p*** -value (Mann–Whitney U Test) *	0.4395	NA	NA
**Prophylactic treatment during the last 4 weeks**			
Patients with record, *n*	41	41	NA
Median (IQR)	0 (0–0)	0 (0–0)	NA
**Without Prophylactic treatment during the last 4 weeks**			
Patients with record, *n*	44	45	NA
Median (IQR)	0 (0–0)	0 (0–0)	NA
***p*** **-value (Mann–Whitney U Test)**	0.4395	0.1648	NA
**With rescue treatment during the last 4 weeks**			
Patients with record, *n*	10	10	NA
Median (IQR)	0 (0–0)	1 (1–1)	NA
**Without rescue treatment during the last 4 weeks**			
Patients with record, *n*	44	45	NA
Median (IQR)	0 (0–0)	0 (0–0)	NA
***p*** **-value (Mann–Whitney U Test)**	0.2614	**<0.0001**	NA
**Asymptomatic form HAE**			
Patients with record, *n*	3	3	3
Median (IQR)	0 (0–0)	0 (0–0)	0 (0–0)
**Mild form HAE**			
Patients with record, *n*	17	18	18
Median (IQR)	0 (0–0)	0 (0–0)	1 (0–3)
**Moderate form HAE**			
Patients with record, *n*	32	32	33
Median (IQR)	0 (0–0)	0 (0–0)	3 (2–7)
**Severe form HAE**			
Patients with record, *n*	2	2	2
Median (IQR)	0 (0–0)	1 (1–1)	12.5 (11–14)
***p*** **-value (Kruskal–Wallis Test)**	0.2180	**0.0469**	**0.0052**

Statistically significant *p*-value (α < 0.05) is highlighted in bold. * Patients with unknown number of acute attacks are not included. NA = not applicable.

**Table 6:** Out-of-pocket cost due to HAE in the last 12 months.

Out-of-Pocket Cost			
	Co-Payments for Healthcare	Co-Payments for Social Care	Increase in Total Costs
Mean (SD) €	0.51 (1.64)	0 (0)	18.28 (71.16)
Median (IQR) €	0 (0–10)	0 (0–0)	0 (0–10)

## Data Availability

The data presented in this study are publicly unavailable due to privacy and ethical restrictions. The data are available on request from the corresponding author M.J.

## Abbreviations

The following abbreviations are used in this manuscript:

AECT	Angioedema Control Test
AE-QoL	Angioedema Quality of Life Questionnaire
C1-INH	C1-inhibitor
HADS	Hospital Anxiety and Depression Scale
HAE	Hereditary Angioedema
QoL	Quality of Life
SD	Standard Deviation
